# Supplementary material for: Treatment-Related Adverse Events in Individuals with BRAF-Mutant Cutaneous Melanoma Treated with BRAF and MEK Inhibitors: A Systematic Review and Meta-Analysis
Source: Cancers (Basel). 2025 Sep 28;17(19):3152. doi: 10.3390/cancers17193152 (PMC12524243; doi:10.3390/cancers17193152)
Supplement: Supplementary file 1 [file cancers-17-03152-s001.zip › Supplementary Table S2.pdf]

Supplementary File S2. Search strategy

Database	Search query	Date of search	Results
**PubMed**	((((("vemurafenib"[MeSH Terms] OR "vemurafenib"[All Fields] OR ("dabrafenib"[Supplementary Concept] OR "dabrafenib"[All Fields]) OR ("encorafenib"[Supplementary Concept] OR "encorafenib"[All Fields]) OR ("trametinib"[Supplementary Concept] OR "trametinib"[All Fields]) OR ("cobimetinib"[Supplementary Concept] OR "cobimetinib"[All Fields]) OR ("binimetinib"[Supplementary Concept] OR "binimetinib"[All Fields]) OR "BRAF inhibitors"[All Fields] OR "MEK inhibitors"[All Fields]) AND "melanoma, cutaneous malignant"[MeSH Terms]) OR "melanoma*"[All Fields] OR "Cutaneous Malignant Melanoma"[All Fields] OR "Cutaneous Malignant Melanomas"[All Fields]) AND "Drug-Related Side Effects and Adverse Reactions"[MeSH Terms]) OR "treatment-related side effect"[All Fields] OR "treatment-related adverse event"[All Fields]) AND "Clinical Trial"[Publication Type] AND 2009/01/01:2025/12/31[Date - Publication]	Feb 2025	395
**Embase**	(('cutaneous melanoma'/exp OR 'cutaneous melanoma' OR (cutaneous AND ('melanoma'/exp OR melanoma))) AND ('braf inhibitor'/exp OR 'braf inhibitor') OR 'mek inhibitor'/exp OR 'mek inhibitor' OR (mek AND ('inhibitor'/exp OR inhibitor))) AND ('treatment related adverse event'/exp OR 'treatment related adverse event') AND [2009-2025]/py	Feb 2025	16
**Scopus**	"BRAF inhibitors" OR "MEK inhibitors" AND "cutaneous melanoma" AND "treatment-related adverse events" AND PUBYEAR > 2011 AND PUBYEAR < 2026	Feb 2025	81

**Cinahl**	"BRAF inhibitors" OR "MEK inhibitors" AND "cutaneous melanoma" AND "treatment-related adverse events"	Feb 2025	319
**Cochrane**	(“BRAF inhibitors” OR “MEK inhibitors” AND “cutaneous melanoma” AND "treatment-related adverse events"):ti,ab,kw (Word variations have been searched) with Cochrane Library publication date Between Jan 2009 and Feb 2025	Feb 2025	220
**Web of Science**	(ALL=(cutaneous melanoma) AND ALL=(bref inhibitor) OR ALL=(mek inhibitor) AND ALL=(treatment-related adverse event)) AND (PY==("2009" OR "2010" OR "2011" OR "2012" OR "2013" OR "2014" OR "2015" OR "2016" OR "2017" OR "2018" OR "2019" OR "2021" OR "2020" OR "2023" OR "2022" OR "2024" OR "2025"))	Feb 2025	92
