# Supplementary material for: Treatment-Related Adverse Events in Individuals with BRAF-Mutant Cutaneous Melanoma Treated with BRAF and MEK Inhibitors: A Systematic Review and Meta-Analysis
Source: Cancers (Basel). 2025 Sep 28;17(19):3152. doi: 10.3390/cancers17193152 (PMC12524243; doi:10.3390/cancers17193152)
Supplement: Supplementary file 1 [file cancers-17-03152-s001.zip › Supplementary Table S3.pdf]

Supplementary File S3. Risk of bias of RCTs using the RoB 2 tool

**Study ID**	**Blinding**	**Outcome**	**Approach to data analysis**	**D1**	**D2**	**D3**	**D4**	**D5**	**Overall**
**Chapman, 2011**	Open-Label	TRAEs (secondary endpoint)	Intention to treat	Low Risk	Low Risk	Low Risk	Some Concern	Low Risk	Some Concern
**Johnson, 2014**	Open-Label	TRAEs (secondary endpoint)	Intention to treat	Low Risk	Low Risk	Low Risk	Some Concern	Low Risk	Some Concern
**Robert, 2015**	Open-Label	TRAEs (secondary endpoint)	Intention to treat	Low Risk	Low Risk	Low Risk	Some Concern	Low Risk	Some Concern
**Dréno, 2017**	Double-Blind	TRAEs (secondary endpoint)	Intention to treat	Low Risk	Low Risk	Low Risk	Low Risk	Low Risk	Low Risk
**Long, 2017**	Double-Blind	TRAEs (secondary endpoint)	Intention to treat	Low Risk	Low Risk	Low Risk	Low Risk	Low Risk	Low Risk
**Maio, 2018**	Double-Blind	TRAEs (secondary endpoint)	Intention to treat	Low Risk	Low Risk	Low Risk	Low Risk	Low Risk	Low Risk
**Dummer, 2018**	Open-Label	TRAEs (secondary endpoint)	Intention to treat	Low Risk	Low Risk	Low Risk	Some Concern	Low Risk	Some Concern
**Robert, 2019**	Double Blind	TRAEs (secondary endpoint)	Intention to treat	Low Risk	Low Risk	Low Risk	Low Risk	Low Risk	Low Risk
**Algazi, 2020**	Open-Label	TRAEs (secondary endpoint)	Intention to treat	Low Risk	Low Risk	Low Risk	Some Concern	Low Risk	Some Concern
**Ferrucci, 2020**	Double Blind	TRAEs (secondary endpoint)	Intention to treat	Low Risk	Low Risk	Low Risk	Low Risk	Low Risk	Low Risk
**Gonzalez Cao, 2021**	Open-Label	TRAEs (secondary endpoint)	Intention to treat	Low Risk	Low Risk	Low Risk	Some Concern	Low Risk	Some Concern
**Daymu, 2024**	Double Blind	TRAEs (secondary endpoint)	Intention to treat	Low Risk	Low Risk	Low Risk	Low Risk	Low Risk	Low Risk

D1: Randomisation process
D2: Deviations from the intended interventions
D3: Missing Outcome data
D4: Measurement of the outcome
D5: Selection of the reported result
