# Supplementary material for: Treatment-Related Adverse Events in Individuals with BRAF-Mutant Cutaneous Melanoma Treated with BRAF and MEK Inhibitors: A Systematic Review and Meta-Analysis
Source: Cancers (Basel). 2025 Sep 28;17(19):3152. doi: 10.3390/cancers17193152 (PMC12524243; doi:10.3390/cancers17193152)
Supplement: Supplementary file 1 [file cancers-17-03152-s001.zip › Supplementary Table S4.pdf]

Supplementary File S4. Risk of bias of prospective cohort studies using the ROBINS-I v2 tool

Study ID	Bias due to confounding	Bias in selection of participants into the study	Bias in classification of interventions	Bias due to deviations from intended interventions	Bias due to missing data	Bias in measurement of outocomes	Bias in selection of the reported result	Overall Bias
**Flaherty, 2012**	High	High	Low	Low	Moderate	Moderate	Moderate	Critical risk of bias
**Kim, 2013**	Moderate	Moderate	Low	Low	Moderate	Moderate	Moderate	Serious risk of bias
**Flaherty, 2014**	Moderate	Low	Low	Low	Low	Low	Low	Moderate risk of bias
**Anforth, 2015**	High	High	Low	Moderate	Moderate	Moderate	Moderate	Critical risk of bias
**Yamazaki, 2015**	High	High	Low	Moderate	Moderate	Moderate	Moderate	Critical risk of bias
**Dika, 2016**	High	High	Moderate	Low	Moderate	Moderate	Moderate	Critical risk of bias
**Schreuer, 2017**	High	High	Low	Moderate	Moderate	Moderate	Moderate	Critical risk of bias
**Si, 2020**	Moderate	Moderate	Moderate	Moderate	Moderate	Moderate	Moderate	Serious risk of bias
**Ribas, 2020**	Moderate	Moderate	Moderate	Moderate	Moderate	Moderate	Moderate	Serious risk of bias
**Nebhan, 2021**	Low	Low	Low	Low	Low	Low	Low	Low risk of bias
**Awada, 2021**	Low	Low	Low	Low	Low	Low	Low	Low risk of bias
**Màrquez-Rodas, 2024**	High	Low	Low	Low	Moderate	Low	Moderate	Serious risk of bias
**Menzies, 2024**	High	Low	Low	Low	Moderate	Low	Moderate	Serious risk of bias
